# Editorial: Photocatalysis and electrocatalysis for energy conversion

**DOI:** 10.3389/fchem.2022.1128243

**Published:** 2023-01-09

**Authors:** Guangzhao Wang, Zhaofu Zhang, Yue-Yu Zhang, Changhong Wang, Kezhen Qi

**Affiliations:** ^1^ Key Laboratory of Extraordinary Bond Engineering and Advanced Materials Technology of Chongqing, School of Electronic Information Engineering, Yangtze Normal University, Chongqing, China; ^2^ The Institute of Technological Sciences, Wuhan University, Wuhan, China; ^3^ University of Cambridge, Cambridge, United Kingtom; ^4^ Wenzhou Institute, University of Chinese Academy of Sciences, Wenzhou, China; ^5^ East China University of Science and Technology, Shanghai, China; ^6^ Hebei Normal University, Shijiazhuang, China; ^7^ College of Pharmacy, Dali University, Dali, Yunnan, China

**Keywords:** photocatalysis, electrocatalysis, optoelectronics, energy conversion, pollution treatment

## Introduction

Photocatalysis and electrocatalysis play important roles in solving energy and environmental problems. Photocatalysis can store solar energy into molecular bonds or utilize solar energy to degrade pollutants by leading various chemical reactions with the help of photocatalysts, while electrocatalysis can implement parallel or similar functions and reactions under external voltage. Recently, the applications of photocatalytic and electrocatalytic technologies in energy and environment fields including hydrogen generation, CO_2_ reduction, O_2_ reduction, and nitrogen fixation have been widely investigated. But the industrial application of photocatalysis and electrocatalysis is still full of challenges, which is mainly limited by the cost and efficiency of current photocatalysts and electrocatalysts. Thus, the design of low-priced and highly efficient photocatalysts and electrocatalysts is still very urgent.

The ideal photocatalysts should possess a long carrier lifetime, a wide light-harvesting region, a strong driving force to carry out oxidation or reduction reaction, while the desired electrocatalysts should utilize very low power energy to drive oxidation or reduction reaction. In this Research Topic “*Photocatalysis and Electrocatalysis for Energy Conversion*,” we have collected 10 articles in total, representing the recent advances in both experimental and theoretical investigations on catalysis, photocatalysis, and optoelectronic devices. Below, we give a brief summary and research highlights about these interesting works.

## Experimental study on catalysis and photocatalysis

Photocatalytic technology could convert solar energy into chemical energy (or clean renewable energy) and degrade pollutants, which is an effective way to solve the energy crisis and environmental pollution. The potocatlaytic activity for hydrogen production of bulk g-C_3_N_4_ is mainly restricted by the fast photoinduced carrier recombination rate, poor visible-light harvest ability, and low specific surface area. Gao et al. summarized the strategies of pH modulation, morphology, control, metal or non-metal dopants, metal deposition, heterojunction or homojunction construction, and dye-sensitization to enhance the photocatalytic performance of pristine g-C_3_N_4_. Sheng et al. prepared two analogous conjugated microporous polymers (CMPs) containing CMP-1 and CMP-2. CMP-1 possesses lower photoexcited carrier recombination than that of CMP-2. Thus, hydrogen production rate of CMP-1 (9,698.53 μmol g^−1^h ^−1^) is about twice of that of CMP-2 (4,727.1 μmol g^−1^h ^−1^). Song et al. prepared a highly efficient Z-scheme g-C_3_N_4_/Ag/AgBr heterostructure photocatalyst, which exhibits excellent photocatalytic activity for tetracycline hydrochloride degradation. Using phosphate organoamine as the structure guiding agent, Ye et al. synthesized an ISAPO-34/SAPO-18 intergrown zeolite. The active temperature window of copper based catalyst prepared from SAPO-34/SAPO-18 shifts to a lower temperature with the increase of copper content. In addition, the Brønsted acid site decreases obviously because of cooper ion exchange and zeolite structure framework damage.

## Theoretical study on catalysis and photocatalysis

First-principles calculations based on density functional theory (DFT) play an increasingly important role in the design of novel catalysts and photocatalysts. Based on DFT theory, Wang et al. predicted that MoWS_4_ monolayer and bilayer are both indirect bandgap semiconductors. Besides, both MoWS_4_ monolayer and bilayer show excellent visible-ultraviolet absorption capacity, and their band edge alignments satisfy the requirement for overall water-splitting. Overall, MoWS_4_ monolayer and bilayer are potential candidates for water-splitting photocatalysts. By utilizing first-principles calculations, Zhang et al. studied the strain effect on the electronic and optical properties of MoTe_2_/PtS_2_ heterostructure. The MoTe_2_/PtS_2_ heterostructure persists the type-II band alignment and the bandgap decreases under external strain. Besides, the compressive strain could tune the band edge positions of MoTe_2_/PtS_2_ heterostructure so as to be suitable for the overall photocatalytic water-splitting at pH 7. Moreover, all the MoTe_2_/PtS_2_ heterostructures show excellent light harvest ability and solar-to-hydrogen efficiency. By calculating and analyzing the electronic and absorptive properties, band edge alignments, Gibbs free energy changes in hydrogen and oxygen evolution reactions, and carrier mobility, Liu et al. predicted ZnO/C_2_N heterostructure to be a promising water-splitting photocatalyst. With the aid of first-principles calculations, Zhang et al. predicted that CdO/HfS_2_ heterostructure is a potential Z-scheme water-splitting photocatlayst, while Han et al. predicted that Fe@χ_3_-borophene is a promising single-atom catalyst for CO oxidation reaction with low energy barrier.

## Novel optoelectronic materials

Yuan et al. predicted MoSSe/InS heterostructure to be an indirect bandgap semiconductor with a type-II band alignment. Biaxial strains could effectively tune the bandgaps, band edge positions, and optical property of MoSSe/InS heterostructure. Besides, the visible-ultraviolet light harvest ability of MoSSe/InS heterostructure is obviously improved as compared with MoSSe and InS single-layers. In general, the MoSSe/InS heterostructure possesses potential application in optoelectronic devices.

We hope this Research Topic could guide new ideas for the search and design of highly efficient catalysts and photocatalysts. Finally, we think all the authors, reviewers, and editors who have contributed to this Research Topic.

